# Ethnicity matters: The case for regional reference intervals in polycystic ovary syndrome (PCOS) diagnostics

**DOI:** 10.17179/excli2025-9011

**Published:** 2026-01-12

**Authors:** Pallav Sengupta, Sulagna Dutta

**Affiliations:** 1 Department of Biomedical Sciences, College of Medicine, Gulf Medical University, Ajman, UAE; 2 Basic Medical Sciences Department, College of Medicine, Ajman University, Ajman, UAE

## ⁯

In recent years, the concept that 'ethnicity matters' in polycystic ovary syndrome (PCOS) diagnostics has shifted from a provocative suggestion to a pressing imperative. The 2023 International Evidence-Based Guideline for PCOS explicitly underscores that '*reference ranges for different methods and laboratories vary widely, and are often based on an arbitrary percentile … of a population that has not been fully characterised*' and recommends that '*normal values should be determined either by the range of values in a well characterized healthy control population or by cluster analysis of general population values*' (Teede et al., 2023[8]). Yet despite this, few clinical settings have translated that ideal into practice, especially in ethnically diverse regions such as MENA or Southeast Asia. Given the deep inter-ethnic variability in anthropometry, body fat distribution, androgen levels, insulin sensitivity, and hirsutism scores, the time has come to demand population-specific cut-offs across *all* diagnostic axes of PCOS, not only for late-stage diagnosis, but especially for early and subtle phenotypes.

First, consider anthropometry and derived indices. It is well established that the same BMI in a South Asian woman corresponds to a higher cardiometabolic risk than in a European woman; waist circumference or waist-to-hip ratio thresholds for metabolic risk differ by ethnicity. These differences should logically carry over to the use of biochemical-anthropometric indices in PCOS risk stratification (e.g. HOMA-IR, or fat-insulin indices). However, current PCOS algorithms largely adopt 'universal' thresholds (e.g. fasting insulin, HOMA-IR cutoffs) derived from European or North American cohorts. This risks both false negatives (in women of 'slender insulin-resistant' phenotypes) and false positives (in ethnic groups with higher baseline insulin). For example, South Asian and Middle Eastern women with PCOS frequently manifest insulin resistance despite lower BMI, and their insulin levels may lie within so-called 'normal' ranges in a European-based reference (VanHise et al., 2023[9]; Baba, 2025[1]). Without regionally calibrated thresholds, early metabolic derangements might be overlooked.

Second, clinical and biochemical hyperandrogenism likewise demand ethnicity-tailored thresholds. The 2023 guideline acknowledges that androgen measurement reference ranges must reflect well characterized control populations and encourages cluster-based derivations (Forslund et al., 2024[5]). But in many regions, androgen assays lack ethnic calibration. The recently published P-PUP (PCOS Phenotype in Unselected Populations) analysis utilized large, medically unbiased, international datasets to derive mFG (modified Ferriman-Gallwey) cutoffs by ethnic cluster: the derived normative cutoffs ranged from 4 to 8, depending on ancestry, and correlate with PCOS features when applied in context (Bizuneh et al., 2025[2]). That same work confirmed that applying a universal cutoff (say 8) may underdiagnose hirsutism in East Asian women or overcall it in populations with robust hair growth norms. In the MENA region and Southeast Asia, where comparative normative mFG data are few, local studies are urgently needed.

Third, ovarian morphology parameters and AMH levels are also potentially modulated by ethnicity. Some studies suggest East Asian women with PCOS more often present polycystic ovarian morphology (PCOM) than Caucasians (e.g. 92.9 % vs 69.9 %) when using standard cutoffs, raising questions whether ultrasound thresholds (e.g. follicle number per ovary, ovarian volume) should be adjusted by ethnicity (Gao et al., 2023[6]). Likewise, AMH normative levels show wide inter-individual and inter-ethnic variation, but most PCOS guidelines adopt 'global' thresholds, risking misclassification in under-represented groups.

Europe provides instructive contrasts: many European or North American cohorts have been the default reference populations, but even within Europe, ethnic minorities (North African, Middle Eastern, South Asian immigration) carry divergent baseline androgen and insulin profiles. Studies from Turkey, for instance, show different mFG norms compared to northern European cohorts (Sendur and Yildiz, 2021[7]). If PCOS diagnosis or risk stratification in Istanbul or Rotterdam fails to adjust thresholds for Turkish or Arab subpopulations, we risk mislabelling or missing disease.

In the MENA region, there is a striking lack of calibrated normative data for PCOS-relevant biomarkers beyond obesity indices. Genetic studies such as the Qatar Genome project illustrate how regionally specific allele frequencies diverge from global references (Fakhro et al., 2016[4]). If reference genomic baselines shift, so might expression levels of steroidogenic or insulin-related genes; this argues for regionally rooted endocrine reference intervals. Unfortunately, many clinical labs in the Gulf still borrow Western normative ranges uncritically.

In Southeast Asia, PCOS burden is rising: a Global Burden of Disease analysis notes steeper increases in age-standardized prevalence and incidence in East and Southeast Asia from 1990 to 2021 (Chai et al., 2025[3]). The region harbors a phenotype of relatively lean but insulin-resistant women. Relying on Western-derived insulin or androgen cutoffs risks deeming many affected women 'normal'. Moreover, East Asian women typically manifest hirsutism at lower androgen thresholds, reinforcing the need for lower ethnic-specific mFG cutoffs (Gao et al., 2023[6]).

Critically, adopting population-specific reference intervals is not an academic luxury but a practical necessity to realize earlier detection. The current diagnostic algorithm often requires two of three criteria; in women whose deviations are mild, failing to capture sub-threshold insulin or androgen elevations can delay diagnosis and intervention until metabolic or reproductive sequelae manifest. Particularly in adolescent or oligomenorrheic women with mild signs, ethnicity-informed thresholds could tip the balance toward earlier recognition and prevention of long-term morbidity.

Inevitably, some will object that implementing multiple regional cutoffs complicates clinical workflows. But the alternative is diagnostic inequity and lost opportunity. Incremental steps are feasible: first, regional or national studies should recruit healthy reference cohorts, cluster-analyze key PCOS biomarkers (insulin, androgens, AMH, mFG) and publish normative intervals stratified by age and BMI strata. Second, laboratories in MENA, Southeast Asia, North Africa and elsewhere should validate or re-calibrate their assay reference ranges accordingly. Third, guideline committees should move beyond recommending 'arbitrary percentiles' toward endorsing calibrated, regionally anchored cutoffs, and explicitly discourage blind adoption of Western norms in underrepresented populations (as the 2023 guideline already gestures toward) (Teede et al., 2023[8]).

Thus, the cry 'ethnicity matters' is not rhetorical, but foundational for equity in PCOS care. Without regionally tailored diagnostic thresholds across anthropometric, biochemical, and morphological dimensions, we risk perpetuating underdiagnosis or misclassification, especially in MENA, Southeast Asia, and non-European ethnicities. As the 2023 guideline correctly envisions, the next frontier in PCOS must be establishment of calibrated, population-specific reference intervals that reflect true biological variation rather than colonial normative hangovers.

## Notes

Pallav Sengupta and Sulagna Dutta contributed equally as first author.

Pallav Sengupta and Sulagna Dutta (Basic Medical Sciences Department, College of Medicine, Ajman University, Ajman, UAE; Phone: +971563973307, E-mail: sulagna_dutta11@yahoo.com) contributed equally as corresponding author.

## Declaration

### Ethical statement

Not applicable

### Conflict of interest

The authors declare no conflict of interest.

### Artificial Intelligence (AI) - assisted technology

The authors used ChatGPT-5.2 (OpenAI) for the sole purpose of improving English language clarity and readability. The conceptualization, scientific content, interpretation of data, and overall manuscript structure were entirely developed by the authors without AI assistance. The authors have reviewed and edited the final text and take full responsibility for the content of this publication.

### Acknowledgments

Not applicable

## References

[1] Baba T (2025). Polycystic ovary syndrome: Criteria, phenotypes, race and ethnicity. Reprod Med Biol.

[2] Bizuneh AD, Joham AE, Tay CT, Kiconco S, Earnest A, Dhungana RR (2025). The PCOS Phenotype in Unselected Populations study: ethnic variation in population-based normative cut-offs for defining hirsutism. Eur J Endocrinol.

[3] Chai O, Feng X, Tang N, Cui D, Li H, Tian W (2025). Global and regional burden of polycystic ovary syndrome, 1990–2021: a systematic analysis of the global burden of disease study 2021. BMC Womens Health.

[4] Fakhro KA, Staudt MR, Ramstetter MD, Robay A, Malek JA, Badii R (2016). The Qatar genome: a population-specific tool for precision medicine in the Middle East. Hum Genome Var.

[5] Forslund M, Melin J, Stener‐Victorin E, Hirschberg AL, Teede H, Vanky E (2024). International evidence‐based guideline on assessment and management of PCOS-A nordic perspective. Acta Obst Gynecol Scand.

[6] Gao Y, Liu H, Qiao L, Liang J, Yao H, Lin X (2023). Study of burden in polycystic ovary syndrome at global, regional, and national levels from 1990 to 2019. Healthcare.

[7] Sendur SN, Yildiz BO (2021). Influence of ethnicity on different aspects of polycystic ovary syndrome: a systematic review. Reprod Biomed Online.

[8] Teede HJ, Tay CT, Laven JJ, Dokras A, Moran LJ, Piltonen TT (2023). Recommendations from the 2023 international evidence-based guideline for the assessment and management of polycystic ovary syndrome. Eur J Endocrinol.

[9] VanHise K, Wang ET, Norris K, Azziz R, Pisarska MD, Chan JL (2023). Racial and ethnic disparities in polycystic ovary syndrome. Fertil Steril.

